# Fast, efficient fragment-based coordinate generation for Open Babel

**DOI:** 10.1186/s13321-019-0372-5

**Published:** 2019-08-01

**Authors:** Naruki Yoshikawa, Geoffrey R. Hutchison

**Affiliations:** 1 Department of Computational Biology and Medical Sciences, Graduate School of Frontier Sciences, The University of Tokyo, Kashiwa, Chiba Japan; 2 Department of Chemistry and Chemical Engineering, University of Pittsburgh, 219 Parkman Avenue, Pittsburgh, PA 15260 USA

**Keywords:** Coordinate generation, Fragments, Molecular geometry

## Abstract

Rapidly predicting an accurate three dimensional geometry of a molecule is a crucial task for cheminformatics and across a wide range of molecular modeling. Consequently, developing a fast, accurate, and open implementation of structure prediction is necessary for reproducible cheminformatics research. We introduce a fragment-based coordinate generation implementation for Open Babel, a widely-used open source toolkit for cheminformatics. The new implementation improves speed and stereochemical accuracy, while retaining or improving accuracy of bond lengths, bond angles, and dihedral torsions. Input molecules are broken into fragments by cutting at rotatable bonds. The coordinates of fragments are set according to a fragment library, prepared from open crystallographic databases. Since the coordinates of multiple atoms are decided at once, coordinate prediction is accelerated over the previous rules-based implementation in Open Babel, as well as the widely-used distance geometry methods in RDKit. This new implementation will be beneficial for a wide range of applications, including computational property prediction in polymers, molecular materials and drug design.

**Electronic supplementary material:**

The online version of this article (10.1186/s13321-019-0372-5) contains supplementary material, which is available to authorized users.

## Introduction

Accurate prediction of the three-dimensional structure of a molecule is critical to a wide range of cheminformatics and molecular modeling tasks, since electrostatic, intermolecular, and other conformation-driven properties depend on the interatomic distances. There is an increasing interest in the “inverse design” of molecules [1] with optimal or near-optimal properties. For example, generative neural networks [2–4] and genetic algorithms [5–7] create molecules with desirable target properties. Moreover, many computational chemistry simulations, including molecular dynamics and quantum chemistry require full three-dimensional structures to run.

Consequently, there have been many proposed methods for three-dimensional coordinate generation, including rule-based, [8, 9] fragment-based [10, 11], and distance geometry embedding methods [12–18]. There are a few free or open source packages capable of coordinatte generation, including BALL [19], FROG [20, 21], RDKit [22], and Open Babel [23]. The latter, which is highly popular, has used a rule-based coordinate builder with a small set of ring fragments, followed by force field minimization. Fragment-based approaches [10] have reported increased accuracy and speed over Open Babel, since no force field minimization is required.

In this work, we discuss an open source implementation of a new fragment-based approach for coordinate generation in Open Babel, with improved accuracy and performance. We compare the stereochemical accuracy with the previous implementation and the open source RDKit distance geometry method, as well as speed and geometric accuracy, measured by heavy-atom root mean square displacement with experimental crystal structures (RMSD), bond distance, bond angle, and torsional/dihedral angle errors. RDKit is chosen as a baseline because it is widely used and a benchmark paper [24] describes it as “competitive with the commercial algorithms”. We also discuss molecules with large geometric or stereochemical errors and future work to improve both geometric and stereochemical accuracy while retaining fast performance.

## Implementation

The new implementation, using a fragment database, required generation of a suitable fragment library, as well as code in Open Babel to perform the new coordinate generation method.

**Fig. 1:**
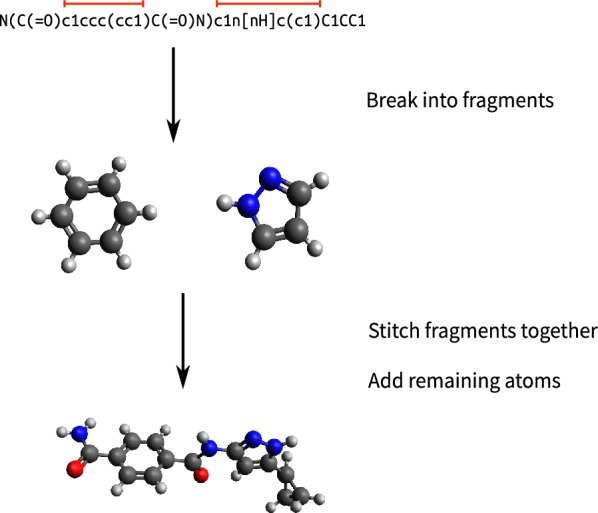
An overview of fragment-based coordinate generation. An input molecule is given without 3D information (e.g. SMILES). The molecule is broken into fragments, which are retrieved from the fragment database. The coordinates of all atoms in a matched fragment are determined in one step, accelerating overall speed of coordinate generation. Fragments and non-fragment atoms are stitched together to generate the final structure

The new fragment-based coordinate generation requires several steps: (1) break the input molecule into fragments, (2) look up fragments from the library, and (3) generate a 3D structure by stitching fragments together. Figure 1 shows an overview of the method. Since fragments include multiple atoms, placement of fragments improves both speed and accuracy over the rule-based method, in which the position of heavy atoms were determined one-by-one.

All figures are produced by Avogadro version 1.2 from the corresponding SDF files [25].

### Generating the fragment library

Before fragment-based coordinate generation can be implemented, a library of known fragments must be created. For this implementation, any molecular substructure which does not have rotatable bonds is considered a fragment. Thus, each molecule is divided into fragments by cutting at all rotatable bonds, including both rings and large non-rotatable functional groups. Small fragments (less than 5 atoms) are not currently stored into the library.

In generating the library of known fragment geometries, we collected 3D structure information from the Crystallography Open Database (COD) [26], the Platinum Dataset [24], and Ligand Expo [27]. We stored only fragments with at least 5 atoms that occurred at least 3 times in the superset of these repositories, creating a total of 5,779 fragments. For each fragment the canonical SMILES was stored—ensuring that only unique substructures were retained. When the same fragment was encountered multiple times, only the first conformation found in the database was stored. Future work will focus on including averaged consensus geometries from similar fragment conformers (e.g., chair vs. twist-boat cyclohexane).

In addition to this main fragment library, the pre-existing Open Babel database of generic ring fragments was retained. This includes $\sim$1000 of the most common ring fragments from analysis of the NCI Open Database [28] and ZINC [29], as well as ring templates from $3-18$ atoms in size, stored as generic SMARTS patterns [30, 31]. This additional library is intended to ensure other ring fragments not explicitly covered in the larger fragment database have approximate matches (e.g., if the stereochemistry or elemental composition differs slightly). Using this auxiliary database is discussed below.

### Breaking down fragments

Coordinate prediction starts by breaking the query molecule into multiple fragments of non-rotatable bonds. For each fragment, the canonical SMILES of each fragment is determined.

### Fragment search

Using the canonical SMILES of a fragment, the coordinates of all atoms in a fragment are retrieved from the flat-file database. To improve performance, an index file is used to determine the file offset of the particular coordinates. The speedup provided by the index is discussed below.

If the exact canonical SMILES is not found in the fragment database, the fragment is tested against the SMARTS patterns of general ring fragments in the auxiliary database. If a fragment is not found in both databases, the atoms are handled by the rules-based atom-by-atom builder. In the set of 4548 Platinum compounds, there were 9741 fragments which have at least five atoms, and 7852 (80.6%) of fragments are found in the COD-based rigid fragment database, 1,887 (19.4%) are partially found in general ring database, and only two fragments are not found in either database.

### Stitching fragments

Building up the entire geometry requires connecting fragments and atom-by-atom rules-based coordinate generation. A working molecule is prepared in the beginning of this process. It only includes atoms of the input molecule, i.e. all bonds are removed. Atoms are iterated by depth-first search order of original molecule. Any atom that has already been determined is skipped from further processing. Otherwise, it is checked for a fragment match. All atoms not matching fragments are connected one-by-one to the working molecule by the previous Open Babel rules-based builder code.

For each fragment match, the geometry of the match is retrieved from the database, and translated to connect to the neighboring atom in the working molecule. The bond vector between the existing atom and the new fragment is determined based on the perceived hybridization of the atom (e.g., sp, sp^2^, sp^3^), the covalent radii of the two elements, and the bond order (i.e., single, double, triple, aromatic).

## Results and discussion

We evaluated the performance of our method on the Platinum dataset, including 4548 organic ligands from the Protein Data Bank [32]. For testing, we used 4432 fragments from the open crystallographic database (COD) to avoid overlap of “training” and test sets. If fragments were drawn from test set molecules, RMSD and other geometric errors would reduce unfairly as fragments know the “answer” of the prediction. For comparison, we considered the most recent release of Open Babel (v. 2.4.1), RDKit (Release 2018.09.1) with the ETKDG method [33] and this new implementation. Each molecule was supplied to the programs as the corresponding SMILES string.

Across the entire set of molecules, we considered the time to generate coordinates and heavy-atom root mean square deviation (RMSD) between the generated and reference molecule. As the RMSD is highly susceptible to differences in conformers, we also considered the mean bond length error, mean bond angle error, and dihedral angle error. Finally, we tested the “success” of retaining the stereochemistry of the original SMILES. All experiments are conducted as single-core processes on a Thinkpad X1 Carbon laptop with Core i7-7500U (2.70GHz), 16GB RAM and Ubuntu 16.04 running on Docker (18.09.1).

**Table 1:** Comparison between implementations

Software	Time (s)	RMSD (Å)	Bond (Å)	Angle ($^\circ$)	Torsion ($^\circ$)	TFD	Success (%)
Open Babel	93.7	1.75	0.055	2.40	48.8	0.27	76.3
RDKit (ETKDG)	274.6	1.59	0.060	2.87	43.9	0.21	99.5
This work	54.8	1.75	0.049	2.49	44.1	0.27	93.9

The performance on 4548 molecules in the Platinum dataset is shown. Time column shows the total time to process all molecules in second. RMSD column shows mean RMSD. Bond, Angle, Torsion columns show mean error of each. TFD column shows mean of the torsion fingerprint deviation [34]. Success indicates the percent of predicted molecules whose InChIKey match that of the original molecule. RMSD and mean error are calculated over successful molecules

**Fig. 2:**
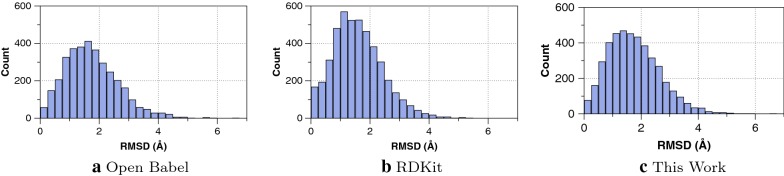
Histograms of heavy-atom RMSD (Å) for **a** the original Open Babel rule-based coordinate generation, **b** RDKit distance geometry method, and **c** current work

As compiled in Table 1 the new implementation improves the stereochemical success rate from 76.3% to 93.9% while dramatically decreasing the time required by almost a factor of two (93.7 s to 54.8 s). The geometric accuracy increases very slightly, in part due to overall decreases in bond and dihedral/torsion errors. The differences between methods in RMSD, bond errors, angles and torsions are all statistically significant through analysis of variance (ANOVA) with p-values less than $1.0\times 10^{-11}$. Illustrated in Fig. 2, the distribution of RMSD across the entire set of 4548 ligands is fairly similar between the original Open Babel implementation, RDKit ETKDG and the new implementation. The relatively high RMSD distribution is largely due to differences in conformations between the stochastic single pose considered here and an ensemble of diverse conformers needed to find low-RMSD geometries.[35–37]

In addition to the mean torsion angle error between the generated geometries and the experimental geometries, we computed the torsion fingerprint deviation (TFD) [34] using RDKit, as an established metric for comparison of torsional errors. The metric ignores hydrogen atoms and minimizes effects of dihedral angles with multiple symmetric atoms. Both the mean torsion angle errors and TFD metrics in Table 1 indicate evident differences in dihedral angles between generated geometries and experimental—the main cause for the relatively high observed RMSD.

The fragment-based method is much faster than other methods because it can determine the coordinates of many atoms at once from the database. Compared to ETKDG, bond length errors and angle errors are generally better (e.g., 0.049 Å vs. 0.060 Å, respectively and 2.49$^\circ$ vs. 2.87$^\circ$, respectively). On the other hand, RMSD and torsion errors are slightly worse than ETKDG, possibly because the current implementation does not consider torsion angle explicitly. Some stereo errors remain, likely because of issues with poor layout of some non-fragment bonds, resulting in incorrect stereochemistry.

**Fig. 3:**
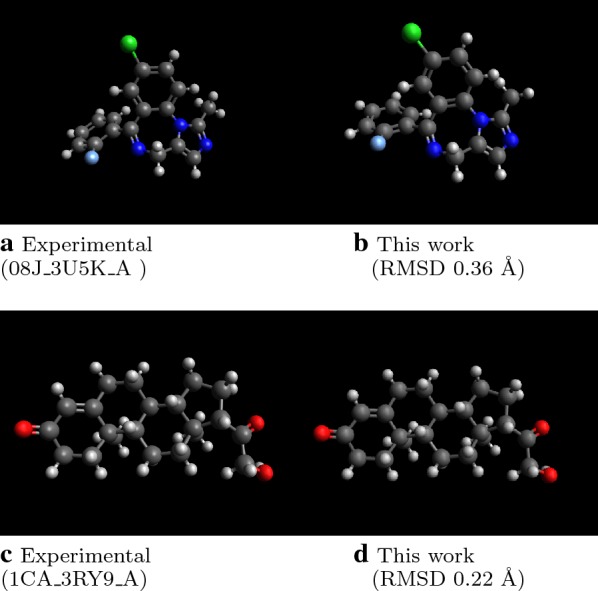
Examples of molecules with low RMSD. Across the molecules in the Platinum set the heavy-atom RMSD between predicted structures and experiment 5.2% falls below 0.5 Å, and 20.5% falls below 1.0 Å, without further force field optimization

Overall, the new implementation is a notable improvement. For example, Fig. 3 indicates two example molecules with very low RMSD to the experimental structure. The processing time is much faster than both released versions of Open Babel and RDKit.

While errors in dihedral/torsion angles exist, the purpose of this study is not to find the conformer that best matches experiment by generating various conformers, but rapidly generating initial geometries for further processing. Some evaluation papers (e.g. [24, 36]) report better RMSD for RDKit or Confab. This is because they generate multiple, geometrically diverse conformers and to find the best RMSD. Such conformer generation is recommended subsequent to creating an initial three-dimensional geometry if desired.[36, 38–45]

### Analysis of problem molecules

**Fig. 4:**
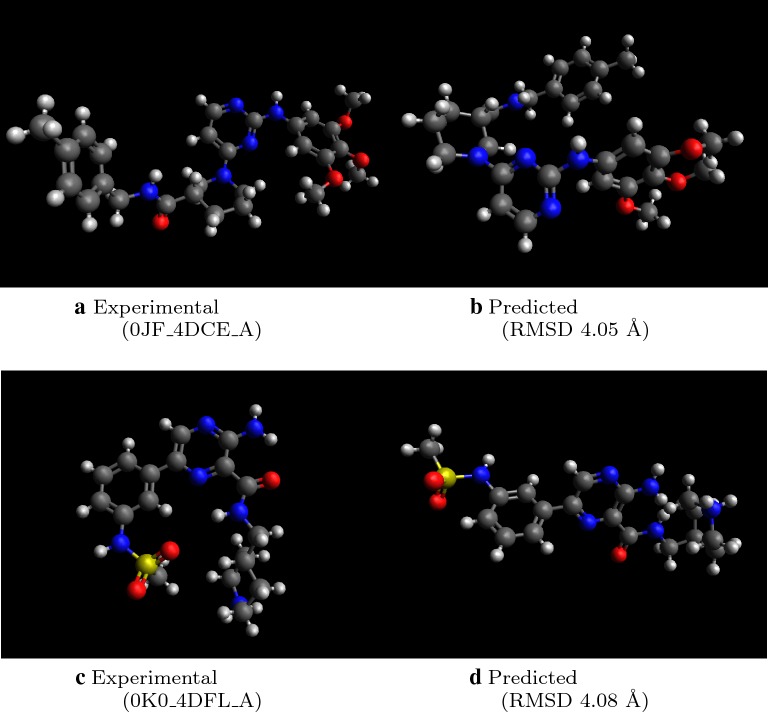
Examples of molecules with high RMSD > 4.0 Å. Note that differences in dihedral angles produce apparently different geometries as judged by RMSD

We find that 9.6% of molecules have RMSD above 3.5 Å or incorrect stereochemistry, compared to 26.8% for the original Open Babel implementation, and only 2.8% for RDKit ETKDG. Figure 4 shows two examples of predicted molecule with high RMSD. In both cases, the main differences between experimental and predicted structures come from inter-fragment dihedral angles. In the bottom example, Fig. 4c, d the predicted conformation is more extended than in experiment. While additional post-processing with conformational searches help to minimize such differences, further work to find patterns of inter-fragment dihedral angle preferences will also improve initial predictions.

**Fig. 5:**
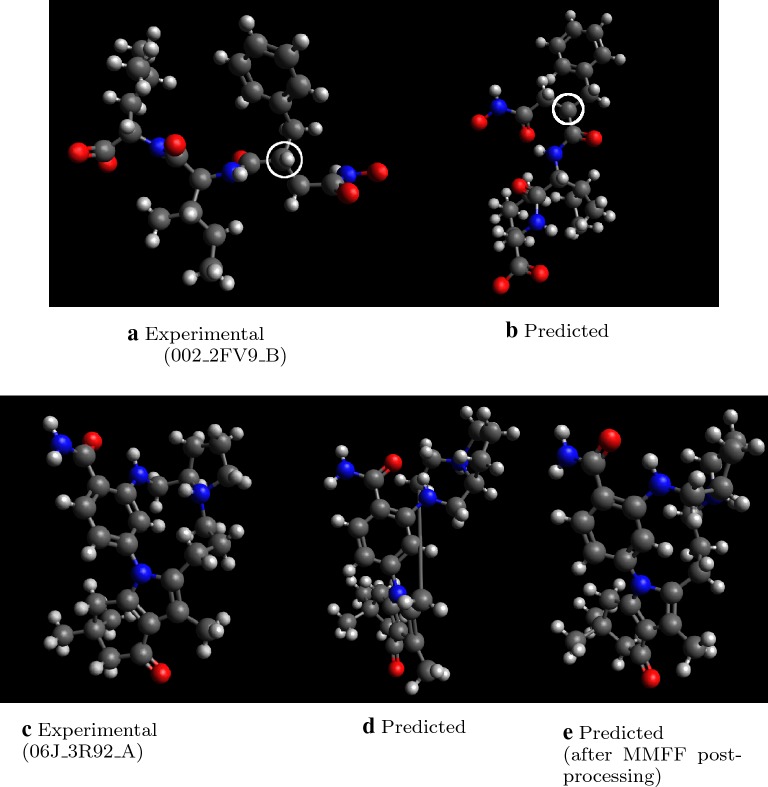
Examples of molecules with incorrect stereochemistry. Note that the bottom case indicates a case with a long ring closure bond in a macrocycle

Beyond poor placement of fragments and choice of dihedral angles, some molecules exhibit incorrect stereochemistry after coordinate generation. Figure 5 shows two examples of molecule with stereochemistry errors. In the first case, an incorrect geometry at the circled carbon yields a difference in stereochemistry after hydrogen atom placement.

In the second case, an extended ring-closure bond occurs in a macrocycle. This structure is generated by stitching small ring fragments together, and they are placed one by one i.e. without considering overall structure. As a result, a long ring-closure bond is inevitable to make ends meet. Post-processing by MMFF optimization (discussed later) reduces these strange connections, but stereochemistry can become scrambled in the process.

As noted above, distance geometry methods such as RDKit ETKDG [33] and stochastic proximity embedding [18] provide greater stereochemistry success rates. The implementation could potentially be improved by using distance geometry to create fragment geometries for species not in the database. Other techniques to augment generation of unusual ring systems and macrocycles should also be explored [46, 47].

### Effects of implementation and post-processing

In order to accelerate the fragment search, we prepared an index file, which stores only the canonical SMILES of fragments and the corresponding position in fragment geometry database. When a match occurs, the file offset of the query fragment is retrieved and the database can be read directly to that position without requiring parsing or searching the entire database. While the latter is currently only $\sim$744 kb in size, as indicated in Table 2 the use of this index file improves performance by a factor of $2.6\times$ (i.e., 140.9 s without the index to 54.8 s with the index).

Beyond the use of the index file, as described above, we also used an auxiliary database of general ring fragments to improve database hit rate. As indicated in Table 2, we tried using the generic fragments before and after placing fragments. Using the generic rings results in better RMSD, in exchange for somewhat longer prediction time (e.g., 20.6 s vs. 54.8 s). More importantly, the generic fragments reduce bond and angle errors and increase stereochemical success. By searching ring fragments before rigid fragments, the prediction accuracy slightly improved but speed and success rate deteriorated. The final implementation places generic ring fragments after exact matching with rigid fragments.

**Table 2:** Effect of implementation

Difference from final	Time (s)	RMSD (Å)	Bond (Å)	Angle ($^\circ$)	Torsion ($^\circ$)	TFD	Success (%)
No index	140.9	1.78	0.056	2.77	45.5	0.30	92.8
No generic rings	20.6	2.01	0.103	4.75	51.5	0.51	89.4
Match generic rings before fragments	56.7	1.78	0.061	2.76	46.9	0.31	92.7
Final implementation	54.8	1.75	0.049	2.49	44.1	0.27	93.9

The performance on 4548 molecules in the Platinum dataset is shown. Time column shows the total time to process all molecules in second. RMSD column shows mean RMSD in Å. Bond, Angle, Torsion columns show mean error of each. TFD column shows mean of the torsion fingerprint deviation [34]. Success indicates the percent of predicted molecules whose InChIKey match that of the original molecule. RMSD and mean error are calculated over successful molecules

We also evaluated the effect of post-processing after coordinate generation. The default –gen3d option for Open Babel performs coordinate generation followed by MMFF94 [48–52] geometry optimization and conformer searching, increasing processing time in favor of producing fewer poor geometries (i.e., incorrectly extended ring closure bonds). As illustrated in Table 3, we find that both methods improve RMSD, bond and angle errors and somewhat increase stereochemical accuracy (to 94.0%) at the cost of $10-20\times$ increased processing time.

The current default Open Babel conformer search is a weighted stochastic rotor search, changing the likelihood of different dihedral angles on the basis of evaluated MMFF94 energies. That is, during the Monte Carlo search, a high-energy conformation will lead to lower likelihood of the associated dihedral angles being chosen in subsequent iterations.

We would generally suggest that users should use both force field optimization and conformer search to reduce bond, angle and RMSD errors, since the resulting processing time is still an average of 0.16s per compound with a single core process on a laptop. This is set as the default option in Open Babel, although users can opt for “fastest” processing if other optimization or conformer search methods are desired.

**Table 3:** Effect of post-processing with MMFF94

Speed	Time (s)	RMSD (Å)	Bond (Å)	Angle ($^\circ$)	Torsion ($^\circ$)	TFD	Success (%)
Fastest (No MMFF)	54.8	1.75	0.049	2.49	44.1	0.27	93.9
Fast (100 MMFF)	372.7	1.72	0.049	2.90	44.1	0.26	93.6
Med (200 MMFF + conf. search)	732.1	1.60	0.048	2.52	43.0	0.23	93.9

The performance on 4548 molecules in the Platinum dataset is shown. Time column shows the total time to process all molecules in second. RMSD column shows mean RMSD in Å. Bond, Angle, Torsion columns show mean error of each. TFD column shows mean of the torsion fingerprint deviation [34]. Success indicates the percent of predicted molecules whose InChIKey match that of the original molecule. RMSD and mean error are calculated over successful molecules

**Fig. 6:**
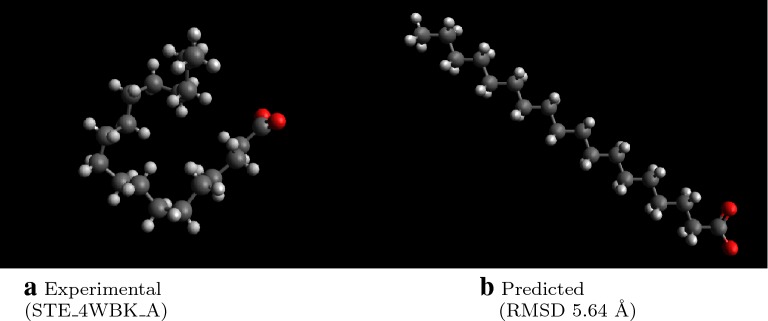
Examples of molecules with high RMSD due to differing dihedral angles. Note that while the experimental conformer reflects a folded alkyl chain, the predicted geometry favors the low-energy extended chain

Despite the geometry optimization and low-energy conformer search, the RMSD remains fairly high. In some cases, this occurs because the lowest energy conformation is not necessarily the same as that in the experimental crystal structure [53]. For example in Fig. 6, the generated structure reflects an extended alkyl chain, the low energy conformation, but the experimental structure is folded. Generating geometrically diverse conformers is one method to find geometries matching such experimental pose. As stated above, performing a thorough conformer search is recommended to find either low-energy structures for modeling or diverse geometries to match experimental conformations found in crystal structures.

Finally, we considered use of a larger fragment database by extracting fragments from all of the COD, Ligand Expo and the Platinum set itself. This larger fragments database includes 5,779 fragments, an additional 1,347 fragments more than the COD-only database, discussed above. Results are compiled in Additional file 1: Table S1. In 9,741 fragments (≥ 5 atoms), 9,004 (92.4%) were found in the rigid fragment database. The number of matches increased from 7,852 (80.6%) in case of using the COD-only database. This increase of matches reduced execution time (54.8 s to 28.1 s, respectively), since more fragments could be placed before searching general ring fragments. However, since almost all fragments were found in either of database even in case of COD-only database, the larger fragment data set did not contribute to increased accuracy.

## Conclusion

We developed a highly efficient open source coordinate prediction method based on a fragment library. The new implementation improves both speed and stereochemical accuracy over the previous rules-based Open Babel implementation. We find that remaining issues often result from missing fragments, resulting in extended ring-closure bonds and in incorrect dihedral angles. Adding explicit rules to handle rare ring fragments and macrocycles will improve these issues. In some cases, molecules with large RMSD to experimental geometries reflect a difference between the low-energy conformer generated by this implementation and the experimental pose, a known problem [53, 54].

We note that increasing the size of the fragment database decreases the generation time, since more atoms can be placed in one step. As an open source, open data method, we anticipate further improvements in the performance of the implementation over time. For example, drawing from sources such as PubChemQC [55, 56] and ZINC [29] will enable incorporating an increasing number of structurally diverse fragments.

Finally, we note that future work, blending this fragment-based method with distance geometry methods (e.g., ETKDG) used by RDKit will combine the speed of fragment-based placement with improved geometric and stereochemical accuracy. As noted above, fragment methods face challenges on less common, macrocyclic, or systems with overlapping fragments. On the other hand, distance-geometry methods face challenges in producing planar aromatic ring systems, which can be found in fragment databases. By combining both methods, we anticipate improved performance across multiple metrics.

## Availability and requirements

- Project name: Open Babel
- Project home page: http://openbabel.org/
- Operating system(s): Platform independent
- Programming languages: C++, Python, Ruby, Java, C#
- Other requirements: Modern C++ compiler
- License: GNU GPL v2.

## Additional file

**Additional file 1.** Histgrams of bond length errors, angle errors, torsion angle errors, torsion fingerprint deviation. Plot of relationship between RMSD and molecular weight, and execution time and molecular weight.

## Data Availability

All the data, including timing logs, SDF files of all 3D coordinates from Open Babel v2.4.1, RDKit, and this implementation, plus evaluation code is available at https://github.com/n-yoshikawa/ob-fragment-generation. The code and fragment database have been successfully merged into the Open Babel development “master” codebase, available at https://github.com/openbabel/openbabel, intended to be part of the future 3.0 release.
