# Supplementary material for: Fast, efficient fragment-based coordinate generation for Open Babel
Source: J Cheminform. 2019 Aug 1;11:49. doi: 10.1186/s13321-019-0372-5 (PMC6676618; doi:10.1186/s13321-019-0372-5)
Supplement: Supplementary file 1 — Additional file 1. Histgrams of bond length errors, angle errors, torsion angle errors, torsion fingerprint deviation. Plot of relationship between RMSD and molecular weight, and execution time and molecular weight. [file 13321_2019_372_MOESM1_ESM.pdf]

Supporting Information

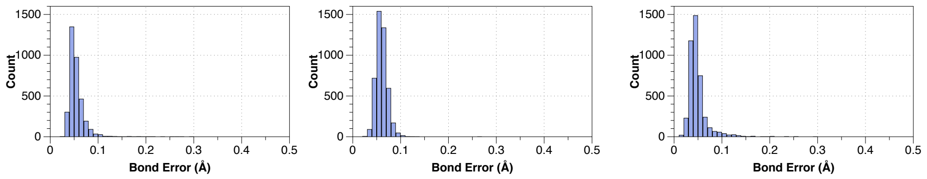

(a) Open Babel (b) RDKit (c) This Work

Figure S1 Histograms of bond length errors (Å) for (a) the original Open Babel rule-based coordinate generation, (b) RDKit distance geometry method, and current work. Note that the new work leaves a fraction of long ring closure bonds.

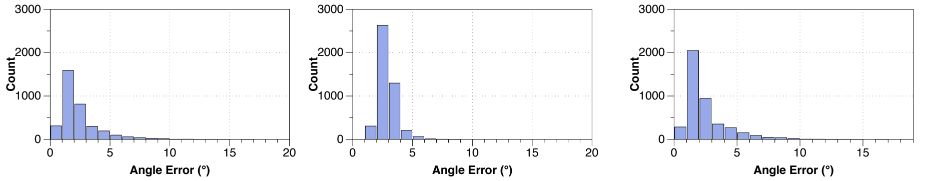

(a) Open Babel (b) RDKit (c) This Work

Figure S2 Histograms of angle errors (°) for (a) the original Open Babel rule-based coordinate generation, (b) RDKit distance geometry method, and current work.

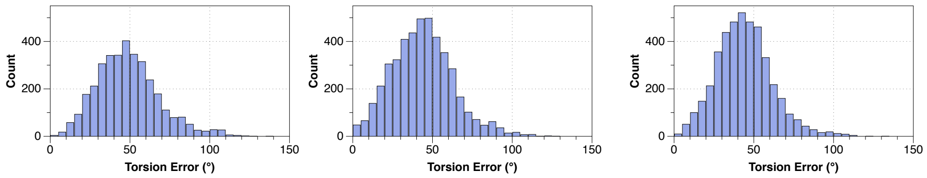

(a) Open Babel (b) RDKit (c) This Work

Figure S3 Histograms of torsion angle errors (°) for (a) the original Open Babel rule-based coordinate generation, (b) RDKit distance geometry method, and current work.

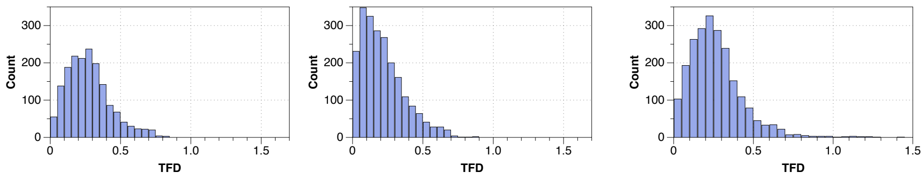

(a) Open Babel (b) RDKit (c) This Work

Figure S4 Histograms of torsion fingerprint deviation (TFD) for (a) the original Open Babel rule-based coordinate generation, (b) RDKit distance geometry method, and current work.

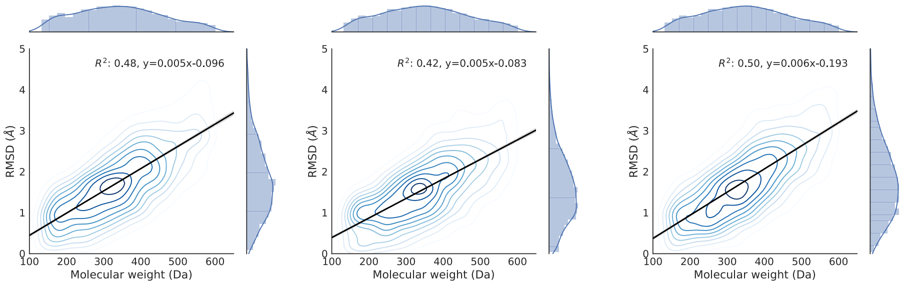

(a) Open Babel (b) RDKit (c) This Work

Figure S5 Relationship between RMSD and molecular weight for (a) the original Open Babel rule-based coordinate generation, (b) RDKit distance geometry method, and current work.

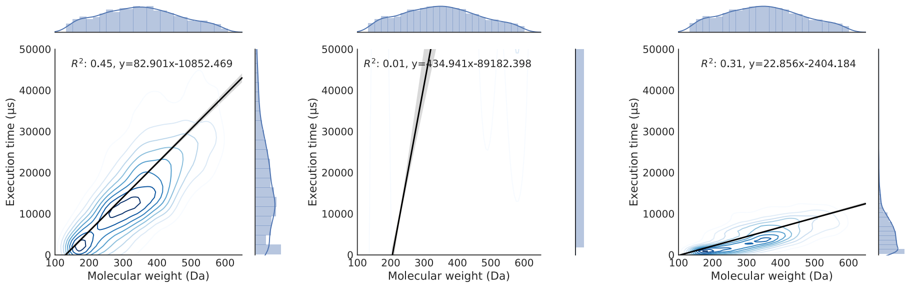

(a) Open Babel (b) RDKit (c) This Work

Figure S6 Relationship between execution time and molecular weight for (a) the original Open Babel rule-based coordinate generation, (b) RDKit distance geometry method, and current work.

Table S1 Using increased fragment database (COD + Platinum). The performance on 4548 molecules in the Platinum dataset is shown. Time column shows the total time to process all molecules in second. RMSD column shows mean RMSD in Å. Bond, Angle, Torsion columns show mean error of each (Å, °, °). TFD column shows mean of the torsion fingerprint deviation [34]. Success indicates the percent of predicted molecules whose InChIKey match that of the original molecule. RMSD and mean error are calculated over successful molecules.

Setting	Time	RMSD	Bond	Angle	Torsion	TFD	Success
Fastest (No MMFF)	28.1	1.75	0.049	2.49	44.1	0.27	93.8
Fast (100 MMFF)	411.9	1.70	0.049	2.75	43.0	0.24	94.1
Med (200 MMFF + conf. search)	744.9	1.58	0.048	2.48	42.3	0.22	94.2
Fastest (COD-only)	54.8	1.75	0.049	2.49	44.1	0.27	93.9
Fast (COD-only)	372.7	1.72	0.049	2.90	44.1	0.26	93.6
Med (COD-only)	732.1	1.60	0.048	2.52	43.0	0.23	93.9
